# Sarcopenia and Associated Factors in Adults Aged 40 and Above: A Study Conducted in Primary Healthcare

**DOI:** 10.7759/cureus.67618

**Published:** 2024-08-23

**Authors:** İrem Şenoymak, Memet T Egici, Mustafa C Şenoymak

**Affiliations:** 1 Family Medicine, Uskudar State Hospital, Istanbul, TUR; 2 Family Medicine, University of Health Sciences, Haydarpasa Numune Training and Research Hospital, Istanbul, TUR; 3 Endocrinology and Metabolism, University of Health Sciences, Sultan Abdulhamid Han Training and Research Hospital, Istanbul, TUR

**Keywords:** primary healthcare services, family medicine, muscle strength, malnutrition, sarcopenia

## Abstract

Introduction: Sarcopenia, characterized by progressive skeletal muscle loss, has emerged as a significant public health concern, with a global prevalence of 10-27%. While traditionally studied in geriatric populations, recent evidence indicates its impact on individuals aged 40 and above, with early manifestations of muscle decline. Primary care settings play a pivotal role in the early identification and management of sarcopenia, facilitating timely diagnosis and intervention. This study aims to evaluate the prevalence of sarcopenia and its associated factors among individuals aged 40 and above attending a family medicine outpatient clinic.

Methods: A cross-sectional study was conducted in a family medicine outpatient clinic, including participants aged 40 and above. Participants underwent handgrip strength measurements, calf circumference measurements, and a 4-m walking test. Based on the criteria of the European Working Group on Sarcopenia in Older People 2 (EGSWOP2), individuals with sarcopenia and those at risk of sarcopenia were identified. The Mini Nutritional Assessment (MNA) was used to assess nutritional status. Sociodemographic characteristics, comorbidities, and laboratory values were recorded. A comparison was made between individuals with normal muscle strength and those at risk of sarcopenia.

Results: Among 213 individuals, 33 (15.4%) were at risk of sarcopenia (probable sarcopenia), and 12 (5.6%) were diagnosed with sarcopenia. There was a negative correlation observed between age and muscle strength (r=-0,339, p<0,001), and positive correlation was found between muscle strength and MNA score (r=0.301, p<0.001). Individuals with higher education and income levels exhibited higher muscle strength. Participants at risk of sarcopenia had higher prevalence rates of comorbidities such as diabetes mellitus (DM), chronic kidney disease, and cardiovascular disease (p=0.20, p<0.01, p=0.015, respectively).

Conclusion: Our study highlights the prevalence and associated factors of sarcopenia in individuals aged 40 and above emphasizing the need for screening and intervention strategies in primary care settings. The study findings support the role of primary care in addressing sarcopenia and improving patient outcomes.

## Introduction

Sarcopenia, a multifactorial syndrome characterized by progressive and generalized skeletal muscle loss, has emerged as a significant public health concern worldwide, with an estimated prevalence % of 10-27. The European Working Group on Sarcopenia in Older People (EWGSOP) was established to provide guidance on the diagnosis, treatment, and management of sarcopenia. In 2018, EWGSOP2 updated its guidelines, offering healthcare professionals direction on the identification, assessment, and management of sarcopenia. EWGSOP2 classifies sarcopenia into three stages. Probable sarcopenia is identified by reduced muscle strength, typically measured by handgrip strength (thresholds: <27 kg for men, <16 kg for women). Confirmed sarcopenia requires both reduced muscle strength and low muscle quantity or quality, assessed through techniques such as dual-energy X-ray absorptiometry (DXA), bioelectrical impedance analysis (BIA), and anthropometry. Severe sarcopenia includes these criteria plus poor physical performance, measured by gait speed (≤0.8 m/s) or other performance tests. This classification aids in early detection and management of sarcopenia, preventing adverse health outcomes [1,2]. It is associated with various adverse health outcomes, including functional decline, increased risk of falls and fractures, frailty, disability, and decreased quality of life [3-6].

While sarcopenia has traditionally been studied in geriatric populations, recent evidence indicates that muscle loss begins in the fourth decade of life. Sarcopenia is prevalent and impactful among individuals aged 40 and above, highlighting the need for early diagnosis and preventive measures [5,7].

Primary care is crucial in health promotion and disease prevention, providing an optimal environment for the early detection and management of sarcopenia. Historically, sarcopenia assessments have been predominantly conducted in hospitalized patients, but primary care settings offer significant potential for these evaluations.

This study aims to contribute to the existing knowledge by evaluating the prevalence of sarcopenia and probable sarcopenia and investigating the associated factors among individuals aged 40 and above attending a family medicine outpatient clinic.

## Materials and methods

Patients and study design

The study was conducted with the approval of the University of Health Sciences Haydarpaşa, Numune Training and Research Hospital Ethics Committee, numbered HNEAH-KAEK2022/173, and in accordance with the Declaration of Helsinki. This cross-sectional descriptive study included volunteer participants who visited the Family Medicine Outpatient Clinic of Haydarpaşa Numune Training and Research Hospital between 29/09/2022 and 30/11/2022. Inclusion criteria for the study were defined as being over 40 years of age, volunteering for participation, and having had the necessary laboratory tests recorded in the health information systems within the past year. The number of individuals over 40 years old who applied to the center where the study was conducted was determined to be an average of 610 over an eight-week period. Retrospective analysis indicated that approximately one-third of these patients had undergone the laboratory tests required for inclusion in the study. Assuming a population of 610 patients, with an estimated 30% eligibility rate for study inclusion, the G*Power (The G*Power Team, Germany) analysis determined that the minimum number of participants needed, with a 95% confidence level and a 5% margin of error, is 211. During the specified dates, a total of 547 patients visited the outpatient clinic, and those aged 40 and above were invited to participate in the study. Individuals unwilling to participate; those who did not wish to complete the Mini Nutritional Assessment (MNA) form or consent to muscle strength and mass measurements; and those with a history of amputation, intellectual disability, unavailable medical data, a diagnosis of primary muscle diseases, or residency in a nursing home or elderly care facility were excluded from the study. All participants included in the study were assessed for sarcopenia by measuring and recording their muscle strength, muscle mass, and physical performance. The MNA was utilized to evaluate the nutritional status of all patients. Sociodemographic information, including smoking and alcohol use, comorbid conditions, and medication use, was obtained through questioning. Subsequently, laboratory data from the year preceding the inclusion of the patients in the study were recorded from the medical records. Participants were categorized into two groups based on handgrip strength measurements using a dynamometer: those with low handgrip strength, indicating probable sarcopenia (risk of sarcopenia), and those with normal handgrip strength, representing the normal group. Laboratory test results and sociodemographic characteristics were compared between the two groups.

Sarcopenia status

“Baseline Hydraulic Hand Dynamometry 2015/NY/USA” was used to measure patients' muscle strength. Three measurements were taken with one-minute intervals between each measurement, and the average of these measurements was calculated and recorded in kilograms [8]. “Probable sarcopenia” or “risk of sarcopenia” was considered when hand grip strength was measured as <27 kg for males and <16 kg for females according to the EWGSOP2 definition [1]. Calf circumference measurements were used to assess muscle mass. A calf circumference measurement of less than 31 cm was defined as low muscle mass for both women and men [9]. Physical performance was assessed through a speed gait test. The participants were instructed to walk at a comfortable pace in a straight, non-slippery area with pre-marked 0 and 4 m indicators. The timing was initiated with the first footstep that crossed the 0 (starting) point, and it was stopped upon hearing the first footstep that crossed the 4-m (finish) line. The average walking time was divided by four to calculate the walking speed in meters per second [10]. A threshold of ≤0.8 m/s was considered as the cutoff for physical performance decline [1]. In the study, the diagnosis of sarcopenia was made according to the diagnostic criteria outlined by EWGSOP2 [1]. Accordingly, patients with probable sarcopenia who had low muscle mass based on calf circumference measurements were diagnosed with sarcopenia. In cases where decreased physical performance was detected during the 4-m walk test in individuals with sarcopenia, this condition was defined as severe sarcopenia.

Nutritional status

The participants' nutritional status was assessed using the MNA questionnaire [11]. The MNA was developed in 1991 and has a sensitivity of 96%, a specificity of 98%, and a positive predictive value of 97% [11]. Validity studies of the MNA have also been conducted in the Turkish population by Sarıkaya et al. [12]. The MNA consists of a total of 18 questions, and scoring is done on a scale of 30 points. A score within 24-30 indicates normal nutritional status, a score between 17-23.5 indicates a risk of malnutrition, and a score below 17 indicates definite malnutrition. The MNA includes measurements of height, weight, upper arm circumference, and calf circumference. The measurements were taken by the same clinician for each patient. After height and weight measurements, body mass index (BMI) was calculated using the formula body weight (kg) divided by the square of height (m^2^).

Statistical analyses

For statistical analysis, SPSS Statistics 23 (IBM Corporation, Armonk, NY) software was used. The normality of quantitative data was assessed using the Kolmogorov-Smirnov and Shapiro-Wilk tests. Descriptive statistical methods, including percentages and mean ± standard deviation (± SD) or median (interquartile range: IQR), were used to describe the data, depending on the normality distribution assessment to ensure the basic characteristics of the data. The chi-square test and Fisher's exact test were used for the comparison of qualitative data. For the comparison of quantitative data, the Mann-Whitney U test was used for non-normally distributed parameters in two-group comparisons, and the independent T-test was used for normally distributed parameters in two-group comparisons. The Kruskal-Wallis test was used for comparisons involving more than two groups for non-normally distributed quantitative variables. Bonferroni correction was applied for pairwise comparisons. The Spearman's correlation test was used for correlation analysis of non-normally distributed quantitative variables. Binary logistic regression models were constructed to identify predictors of risk of sarcopenia. The model included age, sex, and comorbidities (diabetes mellitus (DM), atherosclerotic cardiovascular disease (ASCVD), chronic respiratory diseases (CRD)). A significance level of p<0.05 was considered statistically significant.

## Results

A total of 213 participants were included in the study, consisting of 125 (58.7%) females and 88 (41.3%) males. The age of the participants ranged from 40 to 91 years, with a mean age of 60.42 years. The median height of the participants was 1.64 m, the median body weight was 78 kg, and the median body mass index was 28.30 kg/m^2^.

The majority of participants were married, accounting for 89.7%. Fifty-two percent of the participants were elementary school graduates, while 28.6% were high school graduates. The largest occupation group was housewives (37.6%), followed by retirees (28.6%). The median household size was two people. None of the participants used alcohol, and 73.2% were non-smokers. The socio-demographic characteristics of the participants are shown in Table 1.

**Table 1:** Participants' sociodemographic characteristics ¥ mean±SD † median (interquartile range)

	Participants (n=213)
Age (years)	60.42 ± 10.54 ^¥^
Gender F/M (n,%)	125 (58.7%) / 88 (41.3%)
Height (meters)	1.64 (0.16) †
Body weight (kg)	78 (22) †
BMI (kg/m^2^)	28.30 (5.97) †
Marital status (n,%)	
Married	191 (89.7%)
Other (Single, Divorce, Widow)	22 (10.3%)
Education (n,%)	
Illiterate	13 (6.1%)
Elementary school	111 (52.1%)
High school	61 (28.6%)
University	28 (13.1%)
Occupation (n,%)	
Housewife	80 (37.6%)
Employee	20 (9.4%)
Officer	11 (5.2%)
Self-employment	15 (7%)
Retired	61 (28.6%)
Household	2 (2) ^†^
Smoker (n,%)	
Yes	57 (26.8%)
No	156 (73.2%)

**Figure 1:**
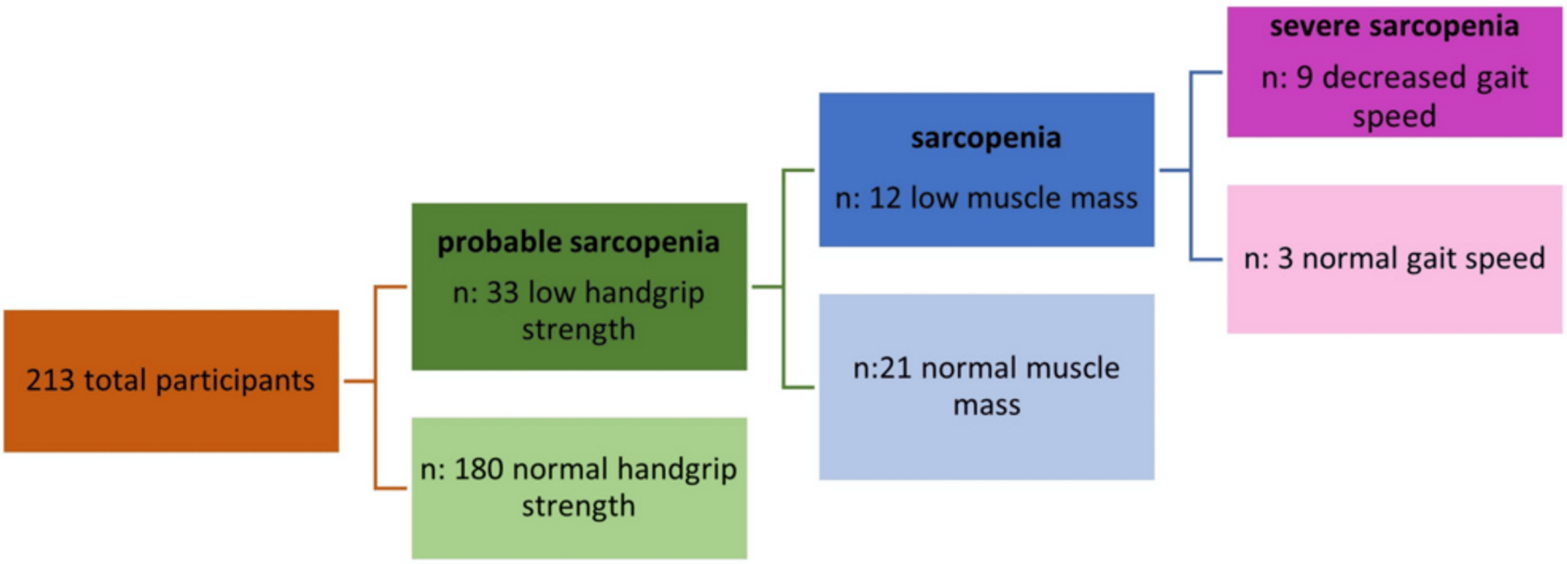
Sarcopenia status of participants

Handgrip strength was found to be normal in 180 individuals, of which 104 (83.2%) were female and 76 (86.3%) were male. There was no significant difference between genders in terms of probable sarcopenia (p=0.530). The participants' sarcopenia status according to the EWGSOP2 criteria is presented in Figure 1.

The most common comorbidities among participants were hypertension (58.7%), type 2 diabetes mellitus (39.4%), cardiovascular disease/atherosclerotic heart disease (27.2%), hyperlipidemia (16.9%), hypothyroidism (16%), asthma/chronic obstructive pulmonary disease (10.3%), chronic kidney disease (5.2%), and cerebrovascular disease (1.4%). Out of the participants, 120 were using antihypertensive medication, 74 were taking oral antidiabetic drugs (OAD), 55 were on antiplatelet therapy, 54 were using proton pump inhibitors (PPI), 47 were taking statins, 44 were receiving vitamin D supplementation, 33 were using insulin, 33 were on selective serotonin reuptake inhibitors (SSRI), 31 were taking levothyroxine, 20 were using B12 supplements, and 14 were taking oral iron.

There was no statistically significant difference between individuals' marital status and handgrip strength (p=0.739). However, a positive statistically significant difference was found between education level, income level, smoking status, and handgrip strength (p<0.001, p=0.008, p=0.008, respectively).

A comparison of comorbidities and probable sarcopenia is shown in Table 2.

**Table 2:** Comparison of comorbidities and risk of sarcopenia ¥ Chi-square test, † Fisher’s exact test DM: Diabetes mellitus; ASCVD: Atherosclerotic cardiovascular disease; COPD: Chronic obstructive pulmonary disease; CRD: Chronic renal disease The statistical significance level was considered as p<0.05.

		Normal (n=180)	Probable Sarcopenia (n=33)	P
Hypertension	Yes (n=125)	101 (80.8%)	24 (19.2%)	0.075 ^¥^
	No (n=88)	79 (89.8%)	9 (10.2%)	
DM	Yes (n=84)	65 (77.4%)	19 (22.6%)	0.020^ ¥^
	No (n=129)	115 (89.1%)	14 (10.9%)	
ASCVD	Yes (n=58)	40 (69%)	18 (31%)	<0.001 ^¥^
	No (n=155)	140 (90.3%)	15 (9.7%)	
Hyperlipidemia	Yes (n=36)	30 (83.3%)	6 (16.7%)	0.831 ^¥^
	No (n=177)	150 (84.7%)	27 (15.3%)	
Hypothyroidism	Yes (n=34)	29 (85.3%)	5 (14.7%)	0.890 ^¥^
	No (n=179)	151 (84.4%)	28 (15.6%)	
Asthma/COPD	Yes (n=22)	17 (77.3%)	5 (22.7%)	0.350 ^†^
	No (n=191)	163 (85.3%)	28 (14.7%)	
CRD	Yes (n=11)	6 (54.5%)	5 (45.5%)	0.015 ^†^
	No (n=202)	174 (86.1%)	28 (13.9%)	

A comparison of the medications used by the participants and probable sarcopenia is shown in Table 3, and risk factors associated with the probable sarcopenia; results of multivariate analysis (binary logistic regression) are shown in Table 4. The logistic regression model was constructed to evaluate factors associated with the risk of sarcopenia. Age (OR (95% CI)=1.09 (1.04-1.14), p<0.001) and ASCVD (OR (95% CI)=2.62 (1.13-6.08), p=0.025) were found to be associated with risk of sarcopenia, after adjustment for age, sex, DM, ASCVD, and CRD (Table 4).

**Table 3:** Comparison of medications used between the groups ¥ Chi-square test, † Fisher’s exact test; PPI: Proton pump inhibitors; SSRI: Selective serotonin reuptake inhibitor The statistical significance level was considered as p<0.05.

		Normal (n=180)	Probable Sarcopenia (n=33)	p-value
Antihypertensive drugs	Yes (n=120)	97 (80.8%)	23 (19.2%)	0.092 ^¥^
	No (n=93)	83 (89.2%)	10 (10.8%)	
Oral diabetic medications	Yes (n=74)	60 (81.1%)	14 (18.9%)	0.313^ ¥^
	No (n=139)	120 (86.3%)	19 (13.7%)	
Antiaggregantes	Yes (n=55)	43 (78.2%)	12 (21.8%)	0.132 ^¥^
	No (n=158)	137 (86.7%)	21 (13.3%)	
PPI	Yes (n=54)	46 (85.2%)	8 (14.8%)	0.873 ^¥^
	No (n=159)	134 (84.3%)	25 (15.7%)	
Statins	Yes (n=47)	32 (68.1%)	15 (31.9%)	<0.001 ^¥^
	No (n=166)	148 (89.2%)	18 (10.8%)	
Vitamin D	Yes (n=44)	39 (88.6%)	5 (11.4%)	0.395 ^¥^
	No (n=169)	141 (83.4%)	28 (16.6%)	
Insulin	Yes (n=33)	21 (63.6%)	12 (36.4%)	<0.001 ^¥^
	No (n=180)	159 (88.3%)	21 (11.7%)	
SSRI	Yes (n=33)	27 (81.8%)	6 (18.2%)	0.642 ^¥^
	No (n=180)	153 (85%)	27 (15%)	
Levothyroxine	Yes (n=31)	27 (87.1%)	4 (12.9%)	0.793 ^†^
	No (n=182)	153 (84.1%)	29 (15.9%)	
Vitamin B12	Yes (n=20)	17 (85%)	3 (15%)	1.000 ^†^
	No (n=193)	163 (84.5%)	30 (15.5%)	
Oral iron supplementation	Yes (n=14)	12 (85.7%)	2 (14.3%)	1.000 ^†^
	No (n=199)	168 (84.4%)	31 (15.6%)	

**Table 4:** Risk factors associated with probable sarcopenia; results of multivariate analysis (binary logistic regression) DM: Diabetes mellitus; ASCVD: Atherosclerotic cardiovascular disease; CRD: Chronic renal disease; OR: Odds ratio, CI: Confidence interval, Number of cases for model: 213; Hosmer-Lemeshow test, p-value = 0.692, Nagelkerke R2 = 0.273, -2 log likelihood= 147.160

	OR	95% CI	p-value
Age (years)	1.09	1.04-1.14	<0.001
Sex	1.71	0.71-4.08	0.228
DM	2.20	0.93-5.20	0.071
ASCVD	2.62	1.13-6.08	0.025
CRD	3.02	0.71-12.89	0.135

The relationship between the laboratory parameters and probable sarcopenia is shown in Table 5.

**Table 5:** Comparison of laboratory and anthropometric parameters between participants with normal and lower handgrip strength ¥ mean ± SD; † median (interquartile range), ‡ Independent T-test, § Mann-Whitney U test BMI: Body mass index; HDL: High-density lipoprotein; LDL: Low-density lipoprotein; NLR: Neutrophil-lymphocyte ratio; TSH: Thyroid-stimulating hormone; FT4: Free thyroxine; CRP: C-reactive protein The statistical significance level was considered as p<0.05.

	Normal (n=180)	Probable Sarcopenia (n=33)	p-value
Age (years)	58.99 ± 9.83 ^¥^	68.18 ± 11.03 ^¥^	<0.001 ‡
Height (meters)	1.64 (0.15) †	1.61 ± 0.10 ^¥^	0.087 §
Weight (kg)	77 (23.5) †	78.39 ± 13.90 ^¥^	0.830 §
BMI (kg/m^2^)	28 (6.07) †	29.20 (6.25) †	0.187 §
Glucose (mg/dL)	101 (28) †	112 (79.5) †	0.137 §
HbA1c (%)	5.95 (1.2) †	7.01 ± 1.57 ^¥^	0.031 §
Creatinine (mg/dL)	0.80 (0.25) †	0.87 (0.38) †	0.328 §
ALT (IU/L)	18 (9.75) †	14 (12) †	0.130 §
AST (IU/L)	18 (6) †	18.58 ± 5.59 ^¥^	0.815 §
Albumin (g/L)	4.48 (0.34) †	4.40 (0.69) †	0.176 §
Total Protein (g/L)	7.10 (0.6) †	6.96 ± 0.48 ^¥^	0.132 §
Sodium (mmol/L)	140 (3) †	139 (2.5) †	0.450 §
Potassium (mmol/L)	4.52 ± 0.45 ^¥^	4.52 ± 0.51 ^¥^	0.983 ‡
Calcium (mg/dL)	9.30 (0.70) †	9.32 ± 0.50 ^¥^	0.995 §
HDL-Cholesterol (mg/dL)	48 (18) †	48.33 ± 14.34 ^¥^	0.881 §
LDL-Cholesterol (mg/dL)	122.35 ± 35.05 ^¥^	113.13 ± 46.29 ^¥^	0.197 ‡
Total-Cholesterol (mg/dL)	202.50 (57.25) †	189.15 ± 58.97 ^¥^	0.246 §
Triglycerides (mg/dL)	128 (82.5) †	143(93) †	0.258 §
Leukocytes (10^3^/mm^3^)	7.21 (2.96) †	7.52 ± 1.94 ^¥^	0.812 §
Hemoglobin (gr/dL)	13.47 ± 1.70 ^¥^	12.90 (2.05) †	0.011 §
Thrombocytes (10^3^/mm^3^)	247 (82) †	247.15 ± 64.95 ^¥^	0.928 §
NLR	1.90 (1.25) †	2.12 (1.05) †	0.357 §
TSH (mIU/L)	1.73 (1.63) †	1.51 (2.23) †	0.926 §
FT4 (ng/dL)	1.14 (0.24) †	1.16 (0.31) †	0.430 §
Vitamin D (ng/mL)	20.55 (13.02) †	19.83 ± 11.33 ^¥^	0.382 §
Vitamin B12 (ng/L)	350 (215) †	370 (357) †	0.387 §
Ferritin (ug/L)	64.05 (83.93) †	84.70 (113.5) †	0.316 §
Folate ( µg/L)	7.40 (4.02) †	7.78 ± 4.21 ^¥^	0.585 §
CRP (mg/L)	2.18 (3.08) †	2.99 (3.55) †	0.164 §

Among the participants in the study, there were 180 participants with a normal MNA score (>24 points), 33 participants at risk of malnutrition, and no participants with malnutrition. A statistically significant positive correlation was found between individuals' muscle strength and MNA score (r=0.301, p<0.001).

The walking speed and MNA scores of participants with and without probable sarcopenia are presented in Table 6.

**Table 6:** The walking speed and MNA scores of participants with and without sarcopenia risk ¥ Chi-square test, MNA: Mini Nutritional Assessment The statistical significance level was considered as p<0.05.

		Probable Sarcopenia (n=33)	Normal (n=180)	p-value
4 meter walk test(speed gait)	Decreased	21 (42%)	29 (58%)	<0.001 ^¥^
	Normal	12 (7.4%)	151 (92.6%)	
MNA	Malnutrition risk	7 (21.2%)	26 (78.8%)	0.323 ^¥^
	Normal	26 (14.4%)	154 (85.6%)	

## Discussion

The present study makes important contributions to the existing knowledge of sarcopenia. It fills an important gap in the literature by focusing on a population often overlooked in research. Additionally, conducting the study in a family medicine outpatient clinic, which offers easy access for individuals from all segments of society, is important for obtaining generalizable and homogeneous results and highlights the role of primary care in addressing sarcopenia. Implementing screening and intervention strategies in such clinics allows for timely diagnosis, risk stratification, and appropriate management, ultimately improving patient outcomes.

In our study, 15.5% of the participants were at risk of sarcopenia, and 5.6% were diagnosed with sarcopenia. The prevalence of sarcopenia varies based on the selected population ranging from 5% to 45% [13-15]. The relatively close prevalence in our study to the lower limits may be attributed to the inclusion of approximately 67% of participants who were under the age of 65 and the recruitment of individuals attending outpatient care. The decrease in muscle mass becomes more pronounced with advancing age [7]. Although the exact pathophysiology of this condition remains incompletely understood, it is believed to be associated with multiple intrinsic and extrinsic factors, including the accumulation of proinflammatory cytokines, oxidative stress, insulin resistance, and loss of motor neuron endplates and anabolic hormones [16,17]. Consistent with the literature, our study also found that participants at risk of sarcopenia and those diagnosed with sarcopenia had higher ages compared to participants without sarcopenia.

In the present investigation, it was found that participants with higher education and income levels had higher muscle strength. This finding is associated with increased awareness through education, a conscious choice of healthy nutrition and exercise, and improved access to adequate nutrition with higher income levels. Similar relationships between income, education level, and nutritional status have been found in studies conducted in the United States [18]. Significantly, studies have shown that lower socioeconomic status correlates with a higher prevalence of sarcopenia, underscoring a major public health issue. Addressing these disparities requires community-based educational programs and initiatives designed to improve access to nutritious food and physical activity opportunities in underserved regions [19,20].

Steffl et al., in their meta-analysis, stated that smoking is an independent factor for sarcopenia; however, they noted contradictory results among the studies, showing both positive and negative correlations between sarcopenia and smoking [21]. The higher muscle strength observed in the smoking group in our study can be explained by the fact that the average age of all participants was 60.4 years, while the average age of the 57 smokers was 55.5 years, which is statistically significant. The duration and amount of exposure to smoking were not evaluated in our study, and studies considering these factors may provide more accurate results.

In our study, the diagnoses of DM, CKD, and ASCVD were significantly higher in individuals at risk of sarcopenia compared to those without sarcopenia. After the logistic regression model was constructed to evaluate factors associated with the risk of sarcopenia, age and ASCVD were found to be associated with the risk of sarcopenia. A meta-analysis has previously reported an increased prevalence of DM in individuals with sarcopenia, attributed to factors such as obesity, physical inactivity, strict dietary practices, potential side effects of medications, accumulation of advanced glycation end products at the cellular level, and the lack of insulin's anabolic effects [22]. Consistent with this, our study also found a significantly higher prevalence of probable sarcopenia among individuals using insulin. Another mechanism could be attributed to poor diabetes regulation and higher levels of glycosylated hemoglobin in patients initiating insulin treatment. In line with this, our study found significantly higher HbA1c values in probable sarcopenic individuals, aligning with the findings of Ai et al.'s meta-analyses [23].

Several studies, consistent with our findings, have shown that sarcopenia is a common condition in patients with CKD. This can be attributed to accelerated protein catabolism, low energy and protein intake, hypoalbuminemia, muscle loss due to uremia and acidosis, as well as increased physical inactivity [24,25].

Studies have also supported a higher prevalence of sarcopenia in individuals with ASCVD compared to the general adult population. Possible reasons for this include increased systemic inflammation, oxidative stress, excessive activation of the ubiquitin-proteasome system, endothelial dysfunction, reduced muscle perfusion, hormonal changes, and physical inactivity [26]. Consistent with the literature, our study found a higher prevalence of sarcopenia in the ASCVD group and among patients using statins. This could be attributed to the potential myopathic effects of statins. Considering the frequent use of statins in patients with ASCVD, it is conceivable that statin use could contribute to the aforementioned possible mechanisms.

Factors contributing to sarcopenia include aging, physical inactivity, nutritional deficiencies, hormonal changes, chronic diseases, genetic factors, smoking, and alcohol use, as well as certain medications, with malnutrition being among the most significant factors. Liguori et al. have found that MNA scores were correlated with muscle strength and mass, and they emphasized the necessity of using MNA for sarcopenia screening [27]. There are limited studies applying MNA to individuals below 65 years old. One such study by Ghazi et al. indicated that MNA is more reliable in elderly individuals and may be more rational to use in younger individuals with chronic diseases [28]. Despite these limitations, considering the well-known association between malnutrition and sarcopenia, we used MNA in our study and observed, found a significant positive correlation between muscle strength and MNA scores, supporting the link between nutritional status and muscle health. The disrupted balance between protein synthesis and breakdown, along with reduced protein intake, likely plays a role, although the exact mechanism is not fully understood [29]. As suggested in previous studies, we believe that these are mutually influencing processes. As nutritional status deteriorates, muscle strength decreases, thereby increasing the risk of sarcopenia. Despite this positive correlation, no significant difference in malnutrition risk based on MNA scores was found between the possible sarcopenic group and the group with normal muscle strength. Possible reasons for this result could be the limited number of probable sarcopenic individuals and the use of MNA with cutoff values determined for this geriatric population [11,12,30].

This study has several limitations. Firstly, the relatively small number of probable sarcopenic individuals limited the robustness of parameter evaluations. Secondly, the use of anthropometric measurements for assessing muscle mass is not considered the gold standard, potentially impacting the accuracy of the results. Additionally, the use of the MNA for nutritional status assessment posed a limitation, as its applicability in individuals below the age of 65 is still being investigated. Moreover, participants were considered to be over 40 years old and were not analyzed separately as geriatric or non-geriatric, which may affect the interpretation of the findings. Despite these limitations, the study has several strengths. It addresses an important age group (40 and above), which is often overlooked in sarcopenia research, thus filling a significant gap in the literature. Conducting the study in a family medicine outpatient clinic increases the generalizability of the results to a broader patient population and highlights the role of primary care in the early identification and management of sarcopenia. The study included a thorough assessment of muscle strength, muscle mass, physical performance, and nutritional status, providing a holistic view of the participants' health status. Additionally, it considered sociodemographic factors such as education, income level, and smoking status, which are important for understanding the broader implications of sarcopenia. To minimize Berkson's bias, we included only outpatients who visited the family medicine clinic. This patient group consists of individuals with generally non-severe health conditions and does not include those requiring hospitalization for serious health issues. This selection aims to represent a broader segment of the population and to minimize sources of bias. By identifying the potential sarcopenic group and examining the relationships with diseases more commonly seen in this group, we aimed to enhance the accuracy of sarcopenia prevalence estimates and improve our understanding of sarcopenia-associated comorbidities. The data collection in a family medicine clinic that is easily accessible to everyone further supports the generalizability of the results to a broader patient population.

## Conclusions

This study highlights the prevalence of sarcopenia and its associated factors among individuals aged 40 and above in a primary care setting, revealing that 15.4% were diagnosed with probable sarcopenia and 5.6% with sarcopenia. Higher education and income levels were positively correlated with muscle strength, while DM, CKD, and cardiovascular disease were more common in those at risk. These findings underscore the importance of early screening and intervention in primary care to improve patient outcomes. Despite its limitations, including small sample size and reliance on anthropometric measurements and the MNA, the study advocates for routine sarcopenia screening in primary care as a significant public health measure. Further research should include larger, more diverse populations and employ gold-standard methods for muscle mass assessment. Additionally, examining the applicability of the MNA in younger populations and those with chronic conditions will help refine diagnostic criteria and intervention strategies for sarcopenia.
